# Rapid evolution of knockdown resistance haplotypes in response to pyrethroid selection in *Aedes aegypti*

**DOI:** 10.1111/eva.13269

**Published:** 2021-07-09

**Authors:** Jennifer Baltzegar, Michael Vella, Christian Gunning, Gissella Vasquez, Helvio Astete, Fred Stell, Michael Fisher, Thomas W. Scott, Audrey Lenhart, Alun L. Lloyd, Amy Morrison, Fred Gould

**Affiliations:** ^1^ Graduate Program in Genetics College of Sciences North Carolina State University Raleigh NC USA; ^2^ Genetic Engineering and Society Center North Carolina State University Raleigh NC USA; ^3^ Biomathematics Graduate Program and Department of Mathematics North Carolina State University Raleigh NC USA; ^4^ Odum School of Ecology University of Georgia Athens GA USA; ^5^ Department of Entomology U.S. Naval Medical Research Unit. No 6. Bellavista Peru; ^6^ Department of Entomology and Nematology University of California Davis CA USA; ^7^ Division of Parasitic Diseases and Malaria Centers for Disease Control and Prevention Atlanta GA USA; ^8^ Department of Entomology and Plant Pathology North Carolina State University Raleigh NC USA

**Keywords:** *Aedes aegypti*, dominance, insecticide resistance, *kdr*, knockdown resistance, selection

## Abstract

This study describes the evolution of *knockdown resistance* *(kdr)* haplotypes in *Aedes aegypti* in response to pyrethroid insecticide use over the course of 18 years in Iquitos, Peru. Based on the duration and intensiveness of sampling (~10,000 samples), this is the most thorough study of *kdr* population genetics in *Ae*. *aegypti* to date within a city. We provide evidence for the direct connection between programmatic citywide pyrethroid spraying and the increase in frequency of specific *kdr* haplotypes by identifying two evolutionary events in the population. The relatively high selection coefficients, even under infrequent insecticide pressure, emphasize how quickly *Ae*. *aegypti* populations can evolve. In our examination of the literature on mosquitoes and other insect pests, we could find no cases where a pest evolved so quickly to so few exposures to low or nonresidual insecticide applications. The observed rapid increase in frequency of resistance alleles might have been aided by the incomplete dominance of resistance‐conferring alleles over corresponding susceptibility alleles. In addition to dramatic temporal shifts, spatial suppression experiments reveal that genetic heterogeneity existed not only at the citywide scale, but also on a very fine scale within the city.

## 1 INTRODUCTION

The mosquito *Aedes aegypti* (L.) transmits yellow fever, dengue, Zika, and chikungunya viruses. Of these viruses, dengue virus (DENV) has the most impact, infecting almost 400 million people annually (Bhatt et al., 2013) and causing an estimated 96 million symptomatic cases and 40,500 deaths every year (GBD 2017 Causes of Death Collaborators, 2018). Although a dengue vaccine is approved in certain parts of the world, the World Health Organization (WHO) recommends that access be restricted by age and prior dengue infection before it can be safely administered (Thomas & Yoon, 2019). Currently, the most common approach to reducing dengue disease incidence continues to be vector control using approaches such as larval habitat source reduction and space or residual surface spraying of chemical insecticides (Dusfour et al., 2019; Ritchie et al., 2021).

Because pyrethroids are highly effective against *Ae*. *aegypti* and exhibit low mammalian toxicity, they have become a preferred tool for *Ae*. *aegypti* population control. Pyrethroids target the voltage‐gated sodium channel protein (VGSC), which is coded for by a single copy gene within the *Ae*. *aegypti* genome (Dong et al., 2014). Due to extensive use, resistance to pyrethroids in *Ae. aegypti* populations is becoming widespread throughout the world (e.g. Bharati & Saha, 2018; Flores‐Suarez et al., 2016; Kamgang et al., 2017; Li et al., 2015; Nguyen et al., 2018; Smith et al., 2016).

Considerable genetic research has been conducted on single nucleotide mutations in the *VGSC* gene that contribute to phenotypic pyrethroid resistance in *Ae*. *aegypti* (reviewed in Du et al., 2016). In Central and South America, pyrethroid resistance in *Ae*. *aegypti* has been associated with single nucleotide mutations that cause amino acid changes from valine (V) to isoleucine (I) at position 1016 (V1016I) and from phenylalanine (F) to cysteine (C) at position 1534 (F1534C) (numbered according to homology in the house fly, *Musca domestica*) in the VGSC protein (Table 1) (Deming et al., 2016; Linss et al., 2014). Another common single nucleotide mutation in Brazil and Mexico that is associated with pyrethroid resistance is the valine (V) to leucine (L) amino acid substitution at locus 410 (Haddi et al., 2017; Saavedra‐Rodriguez et al., 2018). Different nonsynonymous mutations in the *VGSC* gene are more common in other parts of the world. For example, the single nucleotide mutation resulting in the V1016G substitution is common in Asia (Li et al., 2015) and has only recently been reported in the Western Hemisphere (Murcia et al., 2019).

**TABLE 1:** The number of mosquitoes that were genotyped and used for analysis in this study. Total males analyzed are the number of males that were genotyped at both V1016I and F1534C loci and used in the haplotype analysis. Buffer and spray zones correspond to the subset of the total males analyzed that were collected in each area during the 2013 and 2014 suppression experiments. The discrepancy between the totals is due to mosquitoes collected outside of the study area in that year. The V410L genotyped column indicates the subset of total males that were genotyped at the 410 locus in each year

Year	Total Analyzed	Buffer	Spray	V410L Genotyped
2000	628			96
2001	779			103
2002	70			0
2003	389			94
2004	198			92
2005	268			95
2006	796			95
2007	150			6
2008	216			44
2009	433			0
2010	414			98
2011	250			95
2012	400			92
2013	331	252	120	95
2014	3080	1001	1177	70
2015	149			95
2016	809			94
2017	522			92

Because the mutations causing V1016I and F1534C are within the *VGSC* gene, they are expected to be in linkage disequilibrium when polymorphic (Hartl, 2000). Haplotypes that contain both the Cys1534 allele and the wild‐type Val1016 allele are common in wild populations (Deming et al., 2016; Linss et al., 2014). In contrast, the Ile1016 allele is rarely found on the same chromosome as the wild‐type Phe1534 allele (Linss et al., 2014). Vera‐Maloof et al. (2015) suggest that the Cys1534 allele may be required to support the presence of the Ile1016 allele, yet these resistance alleles do not always rise in frequency together. In Brazil, the frequency of both resistance alleles, Ile1016 and Cys1534, increased over time at all sites sampled, but the ratio of different haplotypes of the gene with one or both nucleotide changes varied geographically (Linss et al., 2014). More recently, *Ae*. *aegypti kdr* haplotype analysis has confirmed that the Val1016/Cys1534 haplotype is found worldwide, while the Ile1016/Cys1534 haplotype is found only in the Americas (Cosme et al., 2020; Fan et al., 2020). This implies that both widely dispersed and geographically constrained haplotypes contribute to the widespread pyrethroid resistance observed in *Ae*. *aegypti*.

Although specific resistance haplotypes are commonly observed in *Ae*. *aegypti* in the Western Hemisphere, results from two studies in Merida, Mexico, indicate that heterogeneity in frequencies of common resistance haplotypes can exist at the scale of a city block (Deming et al., 2016). High spatial heterogeneity means that fine‐scale spatial variability is not always observable when assessing insecticide resistance at city or region‐wide scales (Grossman et al., 2019). These two studies are important for understanding the scale of geographic variability in *Ae*. *aegypti* insecticide resistance and the role that limited mobility of this species plays in this evolutionary process. These studies were not, however, able to determine whether the heterogeneity in resistance is due to spatial variation in insecticide pressure or to variation in initial resistance allele frequencies. A better understanding of fine‐scale spatial structure of resistance genetics would help public health officials and scientist preserve the efficacy of important insecticides and aid in the mathematical modeling of new insect control technologies.

Iquitos, Peru, is a well‐established study site for *Ae*. *aegypti* and dengue in the Western Hemisphere with a long history of entomological research and sampling that can help elucidate the factors contributing to pyrethroid resistance evolution in this mosquito (Cromwell et al., 2017; Gunning et al., 2018; LaCon et al., 2014; Morrison et al., 2010). Iquitos is located in the northeastern Peruvian Amazon and is only accessible by boat or plane, and there are no roads into Iquitos. The population was estimated to be ~437,300 in 2015 (Nacional & de Estadistica e Informatica, 2012). *Ae*. *aegypti* were initially eradicated from Peru in 1958, but reinvaded and were detected again in 1984 (Phillips et al., 1992). In anticipation of dengue moving into areas with *Ae*. *aegypti* populations, the U.S. Naval Medical Research Unit No. 6 (NAMRU‐6) established a field office in Iquitos in the late 1980s to monitor for *Aedes*‐transmitted viruses. Because local DENV transmission has occurred since the early 1990s, repeated citywide insecticide spraying and long‐term epidemiological monitoring efforts have been carried out to control *Ae*. *aegypti* populations in an effort to reduce disease (Morrison et al., 2010; Stoddard et al., 2014). Dengue‐1 was detected in Iquitos in 1990 (Phillips et al., 1992), and dengue‐2 was confirmed in 1995 (Watts et al., 1999). Continuous hospital and outpatient clinical surveillance began in 1993 (Watts et al., 1999), and household vector surveillance began in 1998 (Morrison, Gray, et al., 2004). Household vector surveillance collection spans the years before, during, and after pyrethroid use in the city, making Iquitos an excellent location to explore the natural evolution of pyrethroid resistance in *Ae*. *aegypti*.

Here, we examined the temporal and spatial patterns of *kdr* allele frequencies in *Ae*. *aegypti* over 18 years (~180–216 generations) in Iquitos, Peru. This timeframe (2000–2017) spanned years prior, during, and after citywide use of pyrethroids for mosquito control.

A priori, we anticipated that the two most commonly observed *kdr* mutations in Central and South America, V1016I and F1534C, would rise in frequency in response to the application of pyrethroids. We also expected that existing heterogeneity in the population would impact the patterns of resistance haplotype frequencies both temporally throughout the city and spatially on a fine scale among city blocks. In addition, the dominance and selection coefficients of the resistance alleles were anticipated to affect the rate that resistance haplotypes increased in frequency in the population.

## 2 MATERIALS AND METHODS

### 2.1 Entomological surveys

#### 2.1.1 Control history

During the past two decades, researchers have preserved mosquito specimens collected throughout the city of Iquitos in a repository (e.g. Cavany et al., 2020; Cromwell et al., 2017; Getis et al., 2003; Gunning et al., 2018; LaCon et al., 2014; Lenhart et al., 2020; Morrison, Astete, et al., 2004; Morrison, Gray, et al., 2004; Morrison et al., 2006, 2008; Reiner et al., 2019; Schneider et al., 2004; Tun‐Lin et al., 2009). This repository holds samples that were collected prior to pyrethroid application (2000–2002), during citywide pyrethroid use (2002–2014), and after pyrethroids were discontinued by the Ministry of Health (2014–2017). Prior to 2002, no citywide insecticide spraying targeted to control *Ae*. *aegypti* occurred. Once targeted control began, multiple sub‐classes of pyrethroid insecticides were sprayed by the Iquitos Ministry of Health inside homes from 2002 to 2014. These sub‐classes included deltamethrin, cypermethrin, alpha‐cypermethrin, lambda‐cyhalothrin, and alpha‐cypermethrin + pyriproxyfen (Table S1). It was uncommon for residents to spray their own homes (A. Morrison, personal communication). In 2014, pyrethroids were discontinued in favor of malathion due to the development of phenotypic pyrethroid resistance (Gunning et al., 2018). During the years of pyrethroid applications, spraying occurred at an average of 3.25 treatments per year, with spraying occurring within a one‐month period in most years. Because the applications were ultra‐low‐volume space sprays with no residual effect, most generations of the mosquitoes in any year were not exposed to insecticide and mosquitoes were collected independent of whether sprays had been conducted (Ritchie et al., 2021).

#### 2.1.2 Temporal collections

**FIGURE 1:**
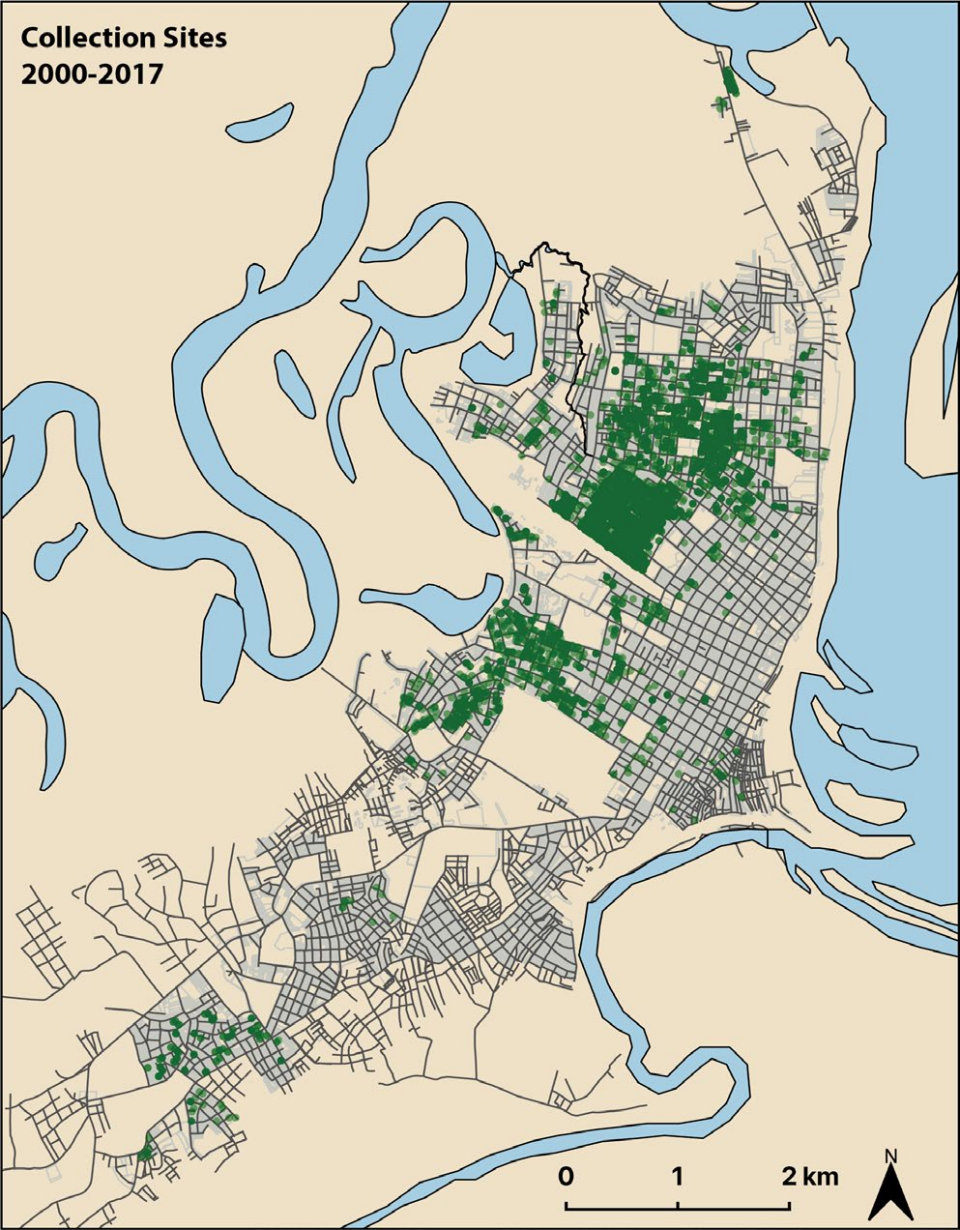
Map of Iquitos, Peru, showing collection sites for *Ae. aegypti* collected from 2000 to 2017. Black lines represent roadways. Collection sites are represented by green circles. Darker green indicates more sampling

*Aedes aegypti* were collected and stored at −80°C by NAMRU‐6 and University of California at Davis personnel since the late 1990s. Specimens dating back to the year 2000 were available for study. Mosquitoes were collected by backpack aspirator (Clark et al., 1994) prior to June 2009 and by Prokopack Aspirator following June 2009 (Reiner et al., 2019; Vazquez‐Prokopec et al., 2009). Each mosquito in the repository was identified to species, sex, collection date, and collection site. Each collection site, typically an individual household, was associated with GPS coordinates (Figure 1).

#### 2.1.3 Spatial collections

**FIGURE 2:**
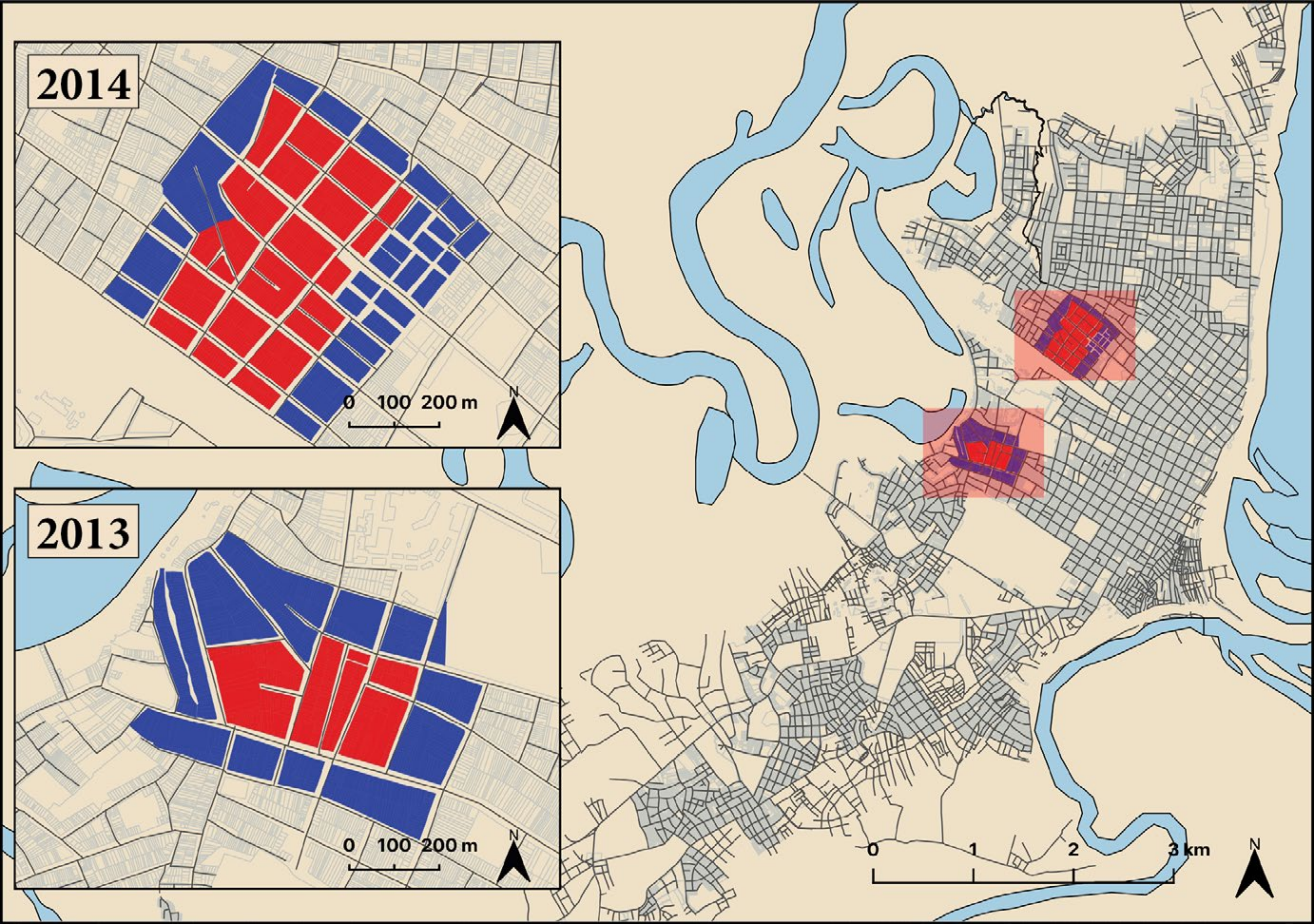
Maps of the 2013 and 2014 suppression experiments showing the buffer (blue) and spray (red) zones from where *Ae. aegypti* were collected. Many houses, often sharing walls, form blocks that are separated by roadways (black lines)

Intense suppression experiments based on pyrethroid spraying were conducted in 2013 and 2014 (Gunning et al., 2018) to test the predictions of a detailed *Ae*. *aegypti* population dynamics model (Magori et al., 2009). In brief, two areas of the city were identified as having relatively high densities of *Ae*. *aegypti* and were configured spatially in a way that allowed for a central spray sector with an outer buffer sector to act as an experimental control region (Figure 2). To limit the impact of migration on resistance allele frequency, site dimensions were selected to be 3–5 times larger than the expected *Ae*. *aegypti* lifetime flight distance of approximately 150 m (Harrington et al., 2005).

The 2013 study site covered approximately 750 m × 450 m and contained 1163 houses. Baseline samples were collected in January 2013. Systematic sampling began on April 22, 2013, and continued for 16 weeks until August 8, 2013. From April 29, 2013, to June 3, 2013, six weekly nonresidual, indoor ultra‐low‐volume (ULV) cypermethrin treatments were applied in the treatment sector.

The 2014 study site was larger and covered an approximate 600 m × 600 m area and contained 2166 houses. Systematic sampling was conducted over a longer period of 44 weeks. ULV spraying of cypermethrin was performed from April 28, 2014, through June 2, 2014, in a similar manner as in the 2013 study. In addition to the study spray in 2014, a citywide spray was conducted in response to a dengue outbreak in February 2014, during which homes in both experimental and buffer sectors were sprayed with pyrethroids. Throughout both suppression experiments, mosquitoes were collected and stored as described above.

### 2.2 DNA extractions and quantification

Male mosquitoes were chosen for genetic analysis throughout this study because female mosquitoes were typically the focus of virology and epidemiological studies, and therefore, more males were available in the repository. Using females would also have brought the risk of genomic contamination from male mosquitoes (via insemination) and from humans (via human blood feeding). Males are expected to share similar allele frequencies with females because the *VGSC* is not sex‐linked.

Whole mosquitoes were transferred from Iquitos, Peru to Raleigh, North Carolina, USA with permits from Peruvian and US authorities. Samples were stored at −80°C prior to genomic DNA (gDNA) isolation and at −20°C after gDNA isolation. Genomic DNA was extracted from whole male *Ae*. *aegypti* by one of two methods: Qiagen DNeasy blood and tissue kit (cat: 69582) or Canadian Center for DNA Barcoding protocol. In brief, for the Qiagen DNeasy kit protocol, whole male mosquitoes were homogenized and incubated in lysis buffer and proteinase K overnight at 55°C. Following incubation and removal of chitinous material, RNase A treatment was performed to remove RNA contamination for both isolation methods. Then, the standard Qiagen protocol of washes was followed. Final samples were eluted two times in 150 µl warm dH_2_O (Invitrogen Cat #: 10977‐015). A modified Canadian Center for DNA Barcoding (2020) protocol was also used for some mosquito DNA isolations to reduce costs while maintaining quality genomic DNA extractions. Samples were homogenized, incubated, and RNase A treated as described above before the lysate was passed through the filter of an AcroPrep™ PALL2 plate (Cat #: PALL 5053) to bind the gDNA. The filter was washed with Protein Wash Buffer to remove remaining proteins and then washed with cold Wash Buffer to remove additional contaminates. The filter was allowed to dry to ensure that no ethanol remained to interfere with DNA yield. Finally, two washes of 75 µl warm dH_2_O (Invitrogen Cat #: 10977‐015) were performed to elute a final volume of 175 µl gDNA.

Quantification of gDNA was performed using a Quant‐iT PicoGreen dsDNA assay (Thermo Fisher Scientific—P11496), and samples were read on a Synergy H1 Hybrid Plate Reader (BioTek Instruments, Inc.) in the Genomic Sciences Laboratory at North Carolina State University (GSL).

### 2.3 Genotyping

Allele‐specific quantitative PCR and melting curve analysis (AS‐PCR) was used to genotype all mosquitoes in duplicate for each of the mutations most commonly found in Central and South America (V1016I and F1534C). If the two reactions were not scored identically, the sample was discarded from further analysis. Mismatches were rare and typically due to nonamplification of a sample or because certain criteria for scoring were not met; that is, melting peak did not cross threshold. A smaller number (*n* = 92) of individuals were additionally genotyped at the V410L locus to verify the strong linkage disequilibrium that has been previously reported between it and locus V1016I (Saavedra‐Rodriguez et al., 2018). Each mosquito genotyped at the V410L locus was also genotyped twice to ensure accuracy.

#### 2.3.1 Genotyping of V1016I

AS‐PCR for the V1016I locus was based on the method reported by Saavedra‐Rodriguez et al. (2007) and modifications to the I1016R primer made by the Entomology Branch at the Centers for Disease Control and Prevention (CDC), Atlanta, USA (A. Lenhart, personal communication). The PCR volume was reduced to 10 µl per reaction and contained 2.5 µl of dH_2_O, 0.5 µl of each primer at 10 µM (V1016F, I1016F, I1016R), 5 µl of PerfeCTa SYBR Green Supermix (Quanta—95054‐02K), and 1 µl of template. The primer sequences for V1016F and I1016F used are reported in Saavedra‐Rodriguez et al. (2007), but the primer sequence for I1016R was modified to: 5’ ‐ TGA TGA ACC SGA ATT GGA CAA AAG C – 3’ (CDC, personal communication). Samples were genotyped on a Bio‐Rad CFX384 Real‐Time PCR machine in the GSL, with the following thermal conditions: step 1—95°C for 3 min, step 2—95°C for 10 s, step 3—60°C for 10 s, step 4—72°C for 10 s, step 5—go to step 2, 39 times, step 6—95°C for 10 s, and step 7—melting curve 65–95°C, increment 0.2°C per 10 s plus a plate read.

#### 2.3.2 Genotyping of F1534C

AS‐PCR for the F1534C locus was performed following the method reported by Yanola et al. (2011) with the following modifications. The PCR volume was reduced to a total of 10 µl per reaction and contained 5 µl dH_2_O, 0.2 µl C1534F primer, 0.4 µl F1534F primer, 0.4 µl F1534R primer, 3.0 µl of PerfeCTa SYBR Green Supermix (Quanta—95054‐02K), and 1 µl of template. Samples were genotyped on a Bio‐Rad CFX384 Real‐Time PCR machine in the GSL, with the following thermal conditions: step 1—95°C for 2 min, step 2—95°C for 30 s, step 3—60°C for 30 s, step 4—72°C for 30 s, step 5—go to step 2, 34 times, step 6—72°C for 2 min, and step 7—melting curve 65–95°C, increment 0.2°C per 10 s plus a plate read.

#### 2.3.3 Genotyping of V410L

The AS‐PCR for the V410L locus was based on a protocol developed by K. Saavedra and shared via the Entomology Branch, CDC (A. Lenhart, personal communication). The total volume for each reaction was reduced to 10 µl: 3.8 µl dH_2_O, 0.05 µl Val410 primer (50 µM) 5’ – GCG GGC AGG GCG GCG GGG GCG GGG CCA TCT TCT TGG GTT CGT TCT ACC GTG – 3’, 0.05 µl Leu410 primer (50 µM) 5’ – GCG GGC ATC TTC TTG GGT TCG TTC TAC CAT T – 3’, 0.1 µl Rev410 primer (50 µM) 5’ – TTC TTC CTC GGC GGC CTC TT – 3’, 5.0 µl PerfeCTa SYBR Green Supermix (Quanta—95054‐02K), and 1 µl template. Thermal conditions were performed on the Bio‐Rad CFX384 Real‐Time PCR machine in the GSL, with the following thermal conditions: 95°C for 3:00, 40 cycles of (95°C for 0:10, 60°C for 0:10, 72°C for 0:30), 95°C for 0:10, and melting curve 65–95°C increasing in increments of 0.2°C per 10 s plus a plate read.

#### 2.3.4 Analysis for AS‐PCR

Melt curve peak calls were determined using the CFX Maestro Software (Bio‐Rad—12004110), verified by eye, and exported to a customized C++ script to quickly convert melt curve peak calls to genotypes for each sample. Melt curve genotypes were then read into a customized R script (R Core Team, 2021) for allele frequency determination and other statistical analysis.

### 2.4 Statistical analysis

#### 2.4.1 Pairwise linkage disequilibrium between SNP markers

Linkage disequilibrium (LD) was calculated between V1016I and V410L for years after emergence using the “genetics” package in R (v. 3.5.0) (Warnes et al., 2019). Pairwise LD was calculated neither between V1016I and F1534C nor between F1534C and V410L because Ile1016 and Leu410 did not appear in the population until Cys1534 was near fixation (see Section 3).

#### 2.4.2 Imputation of haplotypes

**FIGURE 3:**
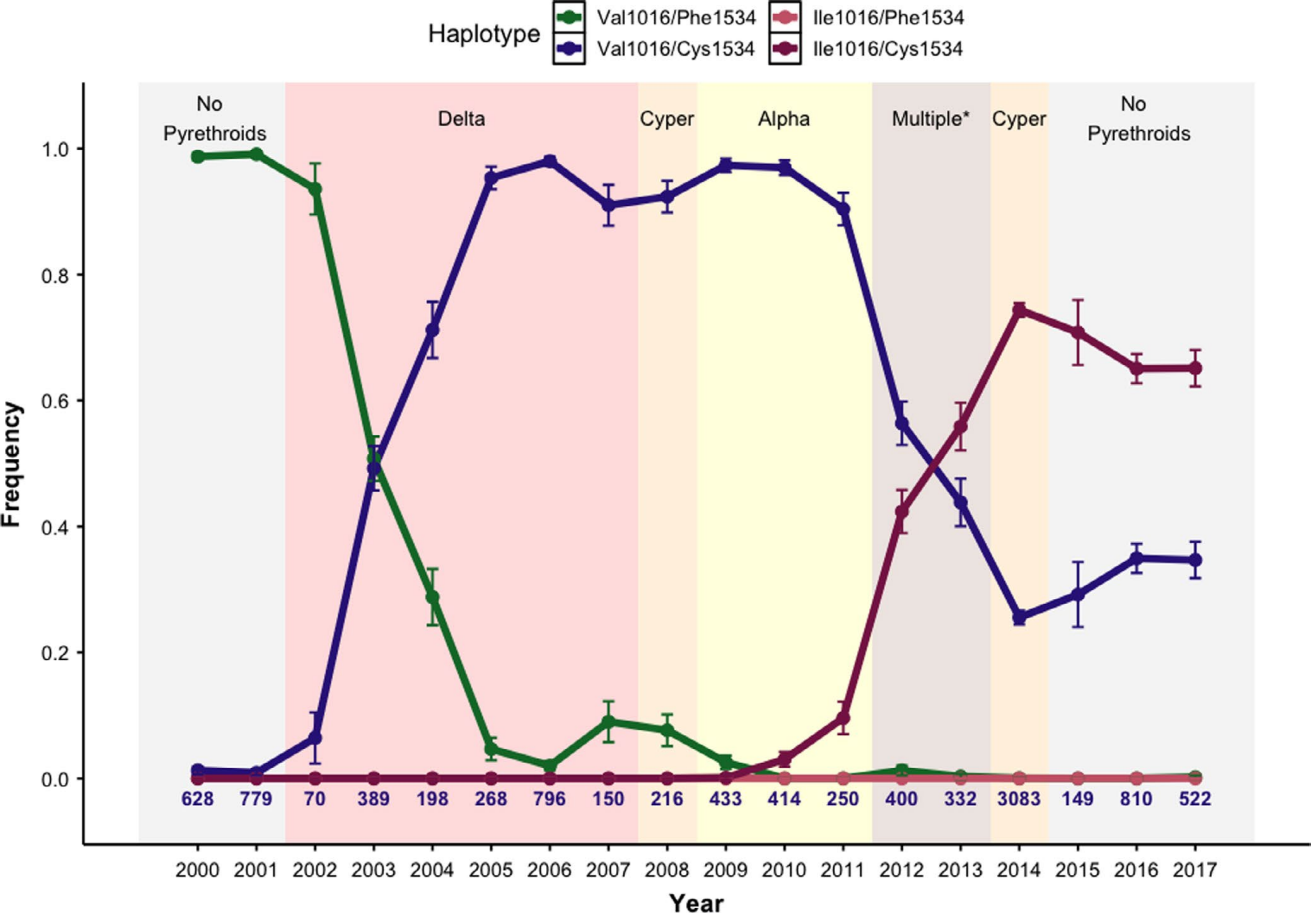
*kdr* haplotype frequencies from 2000 to 2017. Shaded areas represent periods when pyrethroids were used or not. The specific type of pyrethroid is abbreviated in the shaded area (i.e., Delta = deltamethrin, Cyper = cypermethrin, and Alpha = alpha‐cypermethrin). Multiple* represents a period from 2012 to 2013 where multiple chemistries were used, including cypermethrin, alpha‐cypermethrin, lambda‐cyhalothrin, and alpha‐cypermethrin+pyriproxyfen. The number of individuals genotyped per year are shown below *y* = 0.0. Error bars = 95% CI

For individual mosquitoes with at least one homozygous genotype at either locus, haplotypes could be known with confidence. For individual mosquitoes that were heterozygous at either 1534 or 1016 locus, imputed haplotype frequencies were used for analysis because there is a high degree of physical linkage between the SNP loci examined for this study (Linss et al., 2014). To impute the haplotypes, we implemented a custom script in R by first creating a matrix of haplotype probabilities given specific SNP genotypes and considering the assumptions about the existence of the Ile1016/wt1534 haplotype. We then multiplied the genotype count matrix by the probability matrix and summed across the nine genotypes to achieve an estimate of individuals with a given haplotype per year. Finally, haplotype frequencies were calculated from this estimate. For the prior estimate, we assume (1) Mendelian inheritance at both loci and (2) that the resistance allele Ile1016 never occurs with the susceptible allele Phe1534. While this assumption may introduce small errors in the imputation, it is reasonable because the frequency of the Cys1534 allele reaches fixation in the sampled population prior to the emergence of the Ile1016 allele (Figure 3). We observed only 3 out of 9882 individuals (0.03%) which appeared to contain this haplotype (data not shown). Thus, it is likely that few Ile1016/Phe1534 haplotypes existed in the Iquitos population during this study period. Previous investigators have rarely reported instances of this haplotype occurring in Central and South America (Deming et al., 2016; Grossman et al., 2019; Linss et al., 2014; Vera‐Maloof et al., 2015). Vera‐Maloof et al. (2015) reported the largest frequency (0.09), but the frequency decreased in the same area in subsequent collections. The authors concluded that it must have low fitness, even in the presence of pyrethroids.

#### 2.4.3 Statistical analyses

For each year, we conducted a logistic regression to compute confidence intervals on monthly resistance allele frequencies in the spray and buffer zones. We then used these regression models to compute contrast ratios between zones and their corresponding *p*‐values in R via the “emmeans” package (Lenth, 2020). For specific months (October 2002–July 2004) during the increase of the Val1016/Cys1534 haplotype, we tested for Hardy–Weinberg equilibrium (HWE) using the “genetics” package in R (v. 3.5.0) and applied a Bonferroni correction (Warnes et al., 2019).

### 2.5 Parameter estimation of selection coefficient and dominance

#### 2.5.1 Parameter estimation using WFABC

The selection coefficient (s) and dominance (h) of the resistance allele were estimated at each locus in the population for the periods when each resistance allele was increasing in frequency by following the Wright–Fisher approximate Bayesian computation (WFABC) method for temporally sampled data (Foll et al., 2014). Data were input into the model based on the month of collection, and the selection period was set as April 2010–June 2014 and October 2002–December 2009 for locus 1016 and 1534, respectively. The WFABC model can first estimate the effective population size (N_e_) to then use as a prior for determining the selection coefficient, but it assumes a genome‐wide sampling of mostly neutral loci. Because two non‐neutral loci were genotyped, we set 2N_e_ equal to 2 times 500 based on previous research in *Ae*. *aegypti* (Saarman et al. 2017). Saarman et al. (2017) estimated a wide window for N_e_ (25 – 3000), but found that most populations averaged between 400 and 600 for this parameter. We set min_s = −1, max_s = 0, min_h = 0, and max_h = 1. In the WFABC model, genotype fitness assumes a selection benefit to the susceptible allele. Because the susceptible allele was decreasing during the sampled period, we expect a negative selection coefficient. The additional modeling that we implemented, described below, used relative genotype fitness formulas that assumed a selection cost to the susceptible allele. Therefore, we report the absolute value of the WFABC selection parameters to present them in terms of cost to the susceptible allele and to more easily compare results between the WFABC and genotype‐frequency models. In our results, when dominance is zero, the heterozygote behaves exactly like the resistant homozygote and the resistance allele is considered dominant, whereas, when dominance is one, the heterozygote behaves exactly like the susceptible homozygote and the resistance allele is considered recessive.

#### 2.5.2 Parameter estimation using a genotype‐frequency model

We use a genotype‐frequency model to evaluate the spread of the resistance allele at the 1534 locus of the *VGSC*. We assumed the population was well mixed with random mating and that there were discrete (nonoverlapping) generations of mosquitoes, each lasting one month. We also assumed the population was isolated, without immigration or emigration of mosquitoes. These assumptions were used to maintain the simplicity and interpretability of the model.

The full population genetics model (Appendix S1) and sampling distribution form a hidden Markov model (HMM). We conduct Bayesian inference on the HMM using particle Markov chain Monte Carlo (pMCMC). We implement pMCMC with a multivariate normal proposal distribution and adaptive Metropolis–Hastings acceptance in R (R Core Team, 2021) using the package nimble (de Valpine et al., 2017). We used 1000 particles for the particle filter. For parameters in the range [0,1] (*s*, *h*, and the initial frequency, *R*_0_, of the resistance allele), we use uninformative priors of Beta(1,1). For the overdispersion parameter, *A*, we use the uninformative prior *A* ∼ Gamma (0.01, 0.01). To improve the time to convergence, we initialize the parameters using their maximum likelihood estimates. The output of pMCMC is the joint posterior distribution of the parameters along with samples of full time series of genotype frequencies, which we used to construct 95% credible intervals of genotype frequencies.

## 3 RESULTS

### 3.1 Entomological collections

#### 3.1.1 Temporal collections

The number of mosquitoes (Table 1) and sampling areas (Figure 1) varied across years and depend on a number of factors, including where *Ae*. *aegypti* surveys were conducted in any given year and availability of samples. The number of mosquitoes available for use per year ranged from <70 in 2002 to 3080 in 2014. We analyzed a total of 9882 individuals.

#### 3.1.2 Spatial collections

Samples collected in 2013 and 2014 were used to assess spatial variations in allele frequencies because special attention had been paid to the exact location of houses where mosquitoes were collected and the insecticide treatment history of the house (Figure 2). Details can be found in Gunning et al. (2018). Of the total samples collected in 2013 and 2014, 372 males collected in 2013 and 2178 males collected in 2014 were analyzed from the area where the suppression experiments were conducted (Table 1).

### 3.2 Linkage disequilibrium

Linkage disequilibrium between alleles at loci coding for V1016I and V410L was calculated for each year from 2010 to 2017 (Table 2). For all timepoints, LD was significant with a Χ^2^ *p*‐value <0.001 and an *R* ^2^ range of 0.666–0.953. The data indicate that alleles at the two loci were in strong, but not perfect, linkage disequilibrium. Because the *R* ^2^ values were high for alleles at loci V410L and V1016I, the genotype at locus 1016 was used to predict the genotype at locus 410. The wide range in *R* ^2^ values may be attributable to potential relatedness between samples included in the analysis because, for convenience, the samples genotyped at locus V410L were often collected from the same neighborhood.

**TABLE 2:** Linkage disequilibrium between V410L and V1016I from 2010 to 2017 in *Aedes aegypti* collected in Iquitos, Peru

Year	*D*’	*R* ^2^	X^2^	*p*‐value	*n*
2010	0.889	0.666	126.484	<0.001	95
2011	0.999	0.953	148.647	<0.001	78
2012	0.974	0.672	116.942	<0.001	87
2013	1.000	0.802	141.093	<0.001	88
2014	0.979	0.843	114.591	<0.001	68
2015	0.986	0.940	167.359	<0.001	89
2016	0.984	0.742	127.592	<0.001	86
2017	0.966	0.925	170.154	<0.001	92

### 3.3 Temporal *kdr* haplotype frequencies

Prior to the initial use of pyrethroids during 2002 to control *Ae*. *aegypti* in Iquitos, most mosquitoes carried the wild‐type haplotype (Val1016/Phe1534). Immediately after pyrethroid sprays began in 2002, the frequency of the Val1016/Cys1534 haplotype began to rise quickly until it neared fixation in the population in 2006. Notably, the Ile1016/Cys1534 haplotype was not detected until 2010, but then increased in frequency until 2014, when the city stopped spraying pyrethroids and switched to malathion, which has a different mode of action than pyrethroids (Figure 3).

**FIGURE 4:**
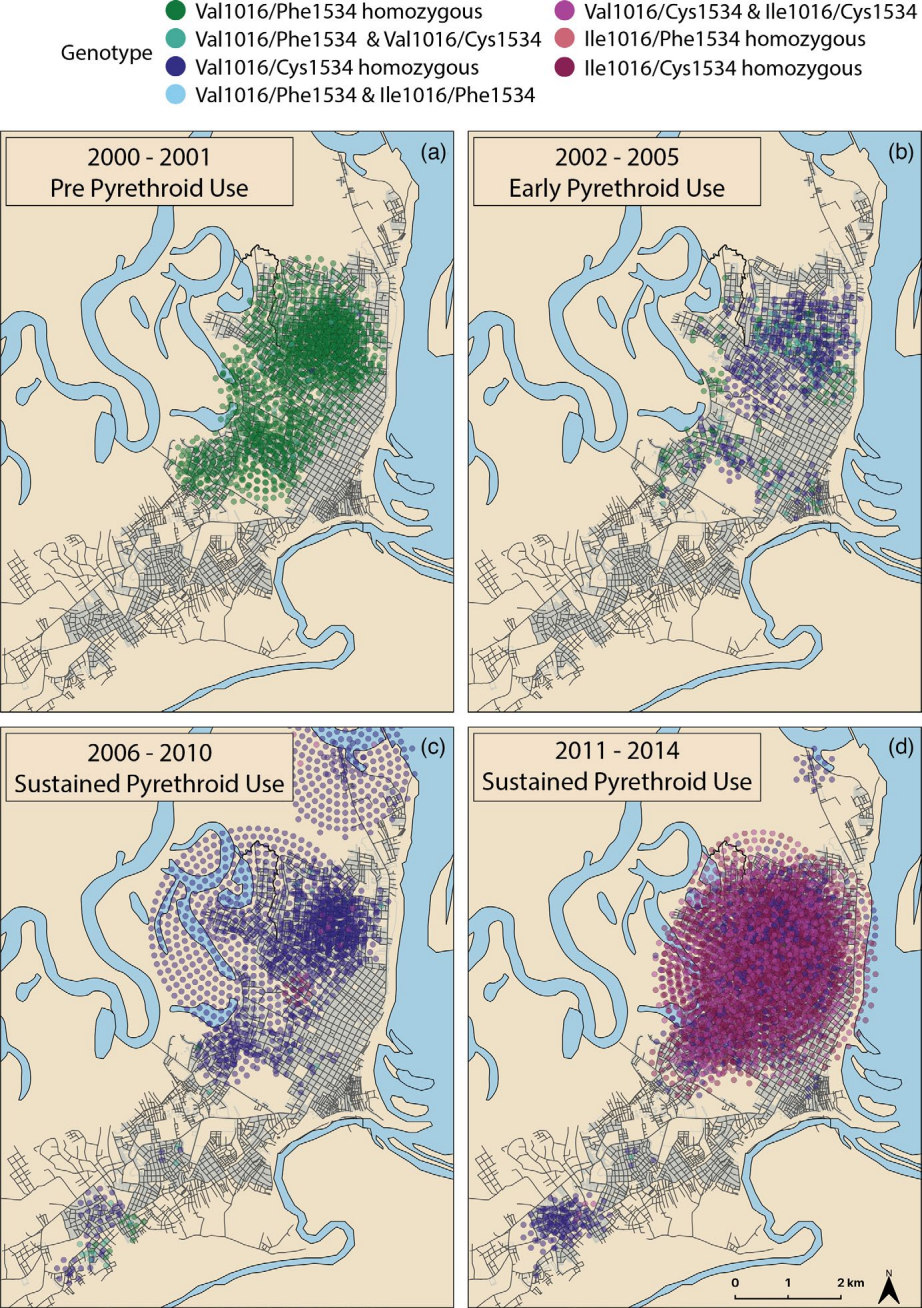
Maps of individual mosquitoes color‐coded by genotype. The colors are similar to the haplotypes depicted in Figure 3. (a) 2000–2001 pre‐pyrethroid use in Iquitos. (b) 2002–2005 early pyrethroid use. (c) 2006–2010 sustained pyrethroid use before Ile1016/Cys1534 haplotype emerges. (d) 2011–2014, sustained pyrethroid use following emergence of the Ile1016/Cys1534 haplotype. Some points are displaced in concentric circles to show individuals collected from the same location

We observed a temporary increase in the wild‐type haplotype (Val1016/Phe1534) frequency in 2007–2008 (Figure 3). The 2007–2008 increase was associated with a spatial expansion of the sampling regime that resulted in the southern part of the city being sampled for the first time (Figure 4c). During this period, a large proportion of *Ae*. *aegypti* in that area of the city carried the wild‐type haplotype. Conversely, the northern part of the city had a higher proportion of haplotypes containing resistance alleles.

**FIGURE 5:**
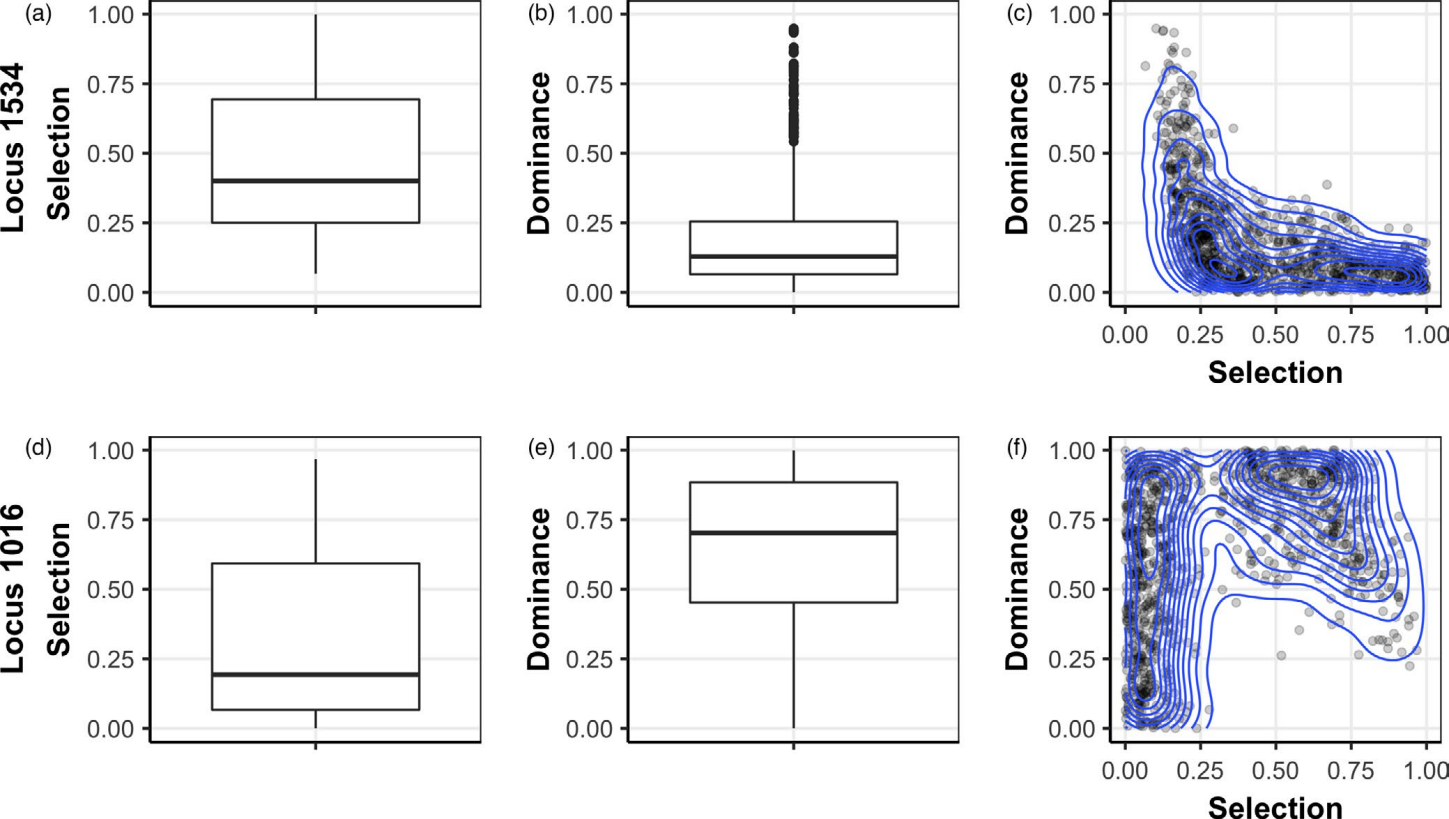
Wright–Fisher approximate Bayesian computation (WFABC) results for locus 1534 (top row) and locus 1016 (bottom row). The box plots are for the estimated selection coefficients (a, d) and dominance (b, e) from 1000 simulated data sets show the median, 1st, and 3rd quartiles, and the whiskers represent the range up to 1.5 times the interquartile range, while all outliers are represented by individual points. The points in the 2D plots (c, f) are joint posteriors estimated by the model, and the blue curves represent contours of the joint posterior distribution

During the time period assessed, we observed large and rapid increases in resistance haplotypes even though citywide, low residual, pyrethroid application only occurred on average 3.25 times per year. Considering the inconsistent selection pressure, the mean evolutionary response per mosquito generation (approx. 4–6 weeks) was strong for the resistance alleles at both loci, albeit confidence intervals were wide. Absolute values of the selection coefficients (95% CI) estimated by the WFABC model were 0.313 (0.007, 0.821) and 0.485 (0.145, 0.969) for loci 1016 and 1534, respectively (Figure 5).

Dominance (95% CI) for each resistance allele was estimated by the WFABC model to be 0.635 (0.057, 0.984) and 0.192 (0.007, 0.702), respectively, for Ile1016 and Cys1534 (Figure 5). Recalling that when dominance is zero, the resistance allele is considered dominant, the results indicate that the Ile1016 resistance allele is partially recessive and that the Cys1534 resistance allele is partially dominant to their respective susceptible alleles. Within each locus, the correlation between estimates of the selection coefficient and dominance is illustrated by the two‐dimensional joint posterior distribution calculated from the WFABC model (Figure 5).

**FIGURE 6:**
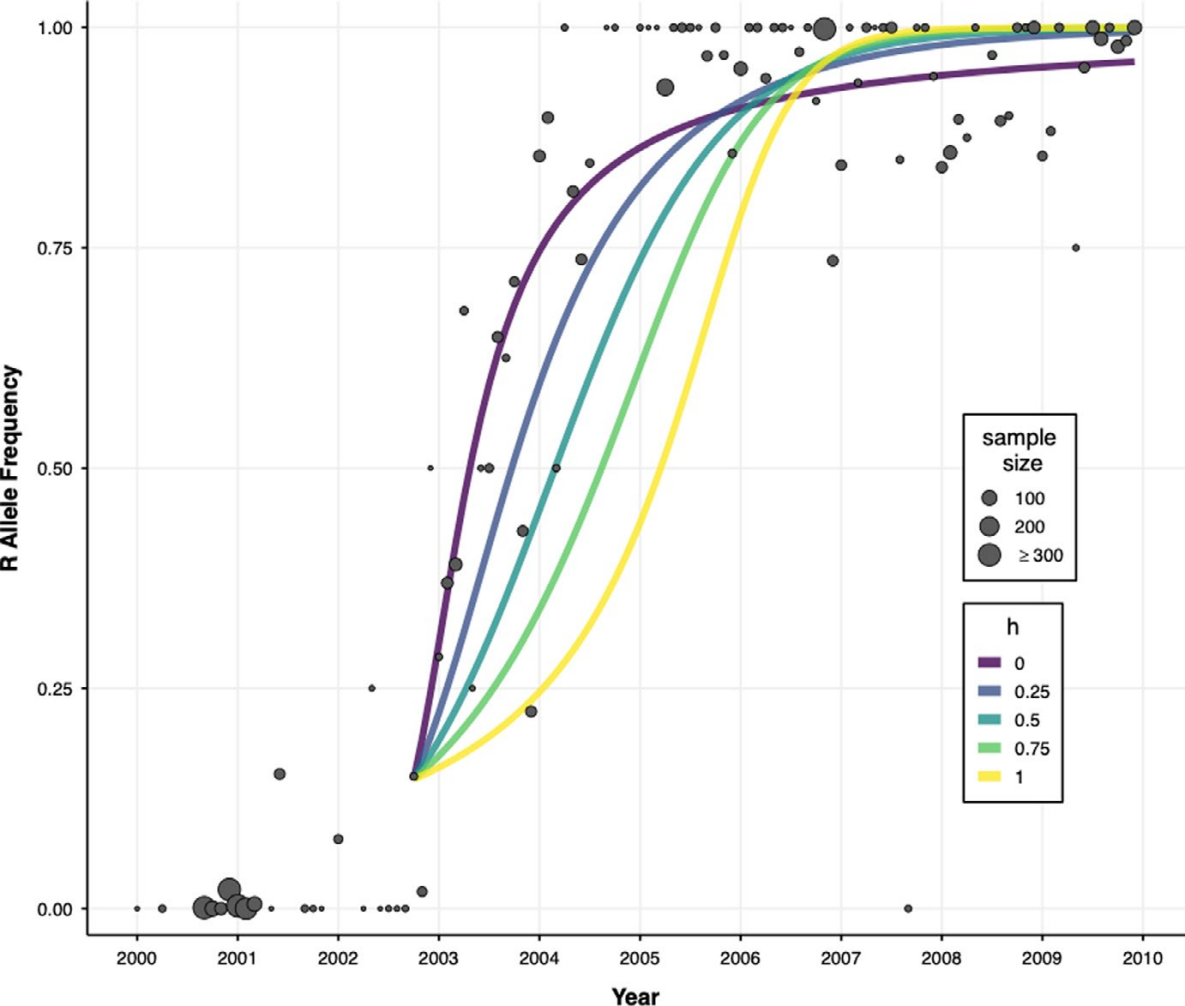
Deterministic model simulations with different dominance parameters. The points indicate empirical 1534 allele (R) frequencies by month, with point size corresponding to the sample size. Model simulations began in October 2002, with initial condition R0 = 0.15 to correspond to the sample of 1 RR, 1 SR, and 8 SS mosquitoes. For each value of h (indicated by color), the value of s providing the maximum likelihood was used for the simulation

Results from our genotype‐frequency model also indicate that the resistance allele, Cys1534, was partially dominant. Population genetic theory and our deterministic simulations indicate that high values of h cause the resistance allele to take longer to increase from low frequencies, but more quickly approach fixation once at high frequencies, while lower values of h result in a quicker initial increase in frequency, but a longer time to fixation (Figure 6). The observed dynamics in our system appear to be captured better with lower values of h in the simulations (Figure 6 and Figure S1). Using pMCMC to estimate parameters of the system, the mean (95% credible interval) for dominance and selection coefficient was *h* = 0.184 (0.011, 0.571) and *s* = 0.188 (0.087, 0.284), respectively. The estimated partial dominance of the resistance allele reflects the behavior of the deterministic simulations, though there is a high degree of uncertainty. Additionally, the estimates for the initial resistance allele frequency and overdispersion parameter were *R*_0_ = 0.270 (0.135, 0.450) and *A* = 4.91 (2.97, 7.43). The value for *R_0_* was determined by selecting the resistance allele frequency in October 2002, which is the month that pyrethroid spraying most likely began in earnest in Iquitos. The joint posterior distributions demonstrate correlation between parameter estimates, with a particularly strong correlation between estimates of *h* and *s* and between *R_0_* and *s* (−0.670 and −0.752, respectively), as well as between *h* and *R_0_* (0.536) (Figure S2). For example, without large samples in the first several generations, it may not be possible to distinguish a low initial frequency and large fitness cost from a higher initial frequency but lower cost. We also used the genotype‐frequency time‐series samples from pMCMC to construct mean genotype estimates and 95% credible intervals (Figure S3). In later years, these genotype estimates suggest that the susceptible allele is maintained in the population in heterozygotes, which is a result of the low estimates for *h*.

We noticed that our pMCMC model appeared to predict a greater abundance of heterozygotes than were observed in the empirical data during the period when the Val1016/Cys1534 haplotype was increasing (Figure S3). However, the HWE analysis shows only three months (January 2003, March 2003, and December 2003) where our observed genotype frequencies were not in HWE (Table S2).

### 3.4 Spatial *kdr* haplotype frequencies

**FIGURE 7:**
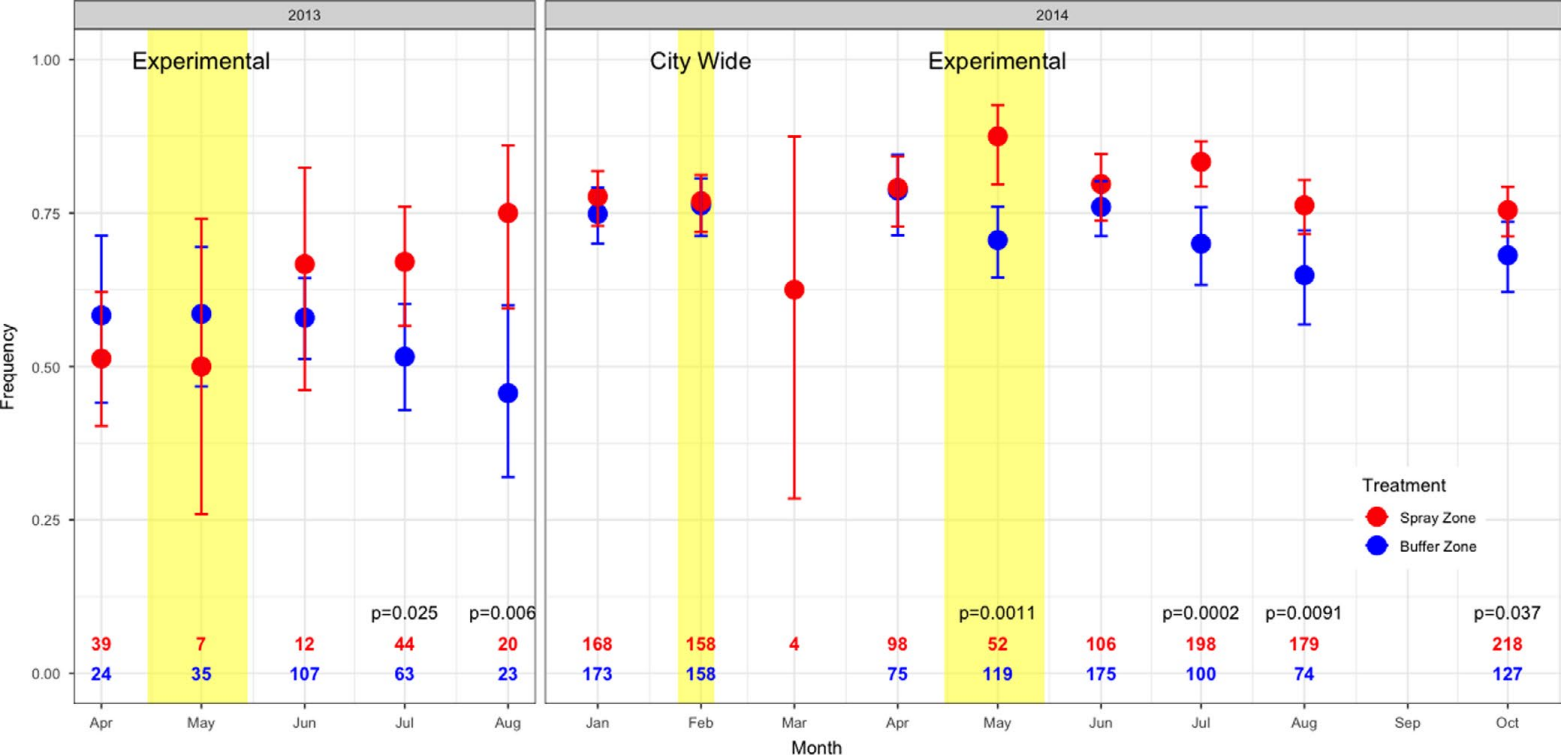
Frequency of Ile1016/Cys1534 haplotype by treatment group across the year 2013 (left) and 2014 (right). A 6‐week experimental cypermethrin application, highlighted with yellow blocks, occurred in the spray zone from April 29 to June 3, 2013, and from April 28 to June 2, 2014. A citywide cypermethrin spray in response to a dengue outbreak occurred in February 2014 and is also highlighted with a yellow block. *p*‐values for a test of equality between treatment groups by month were computed from generalized linear models (shown for only for *p* < 0.1)

During 2013 and 2014, the haplotype Ile1016/Cys1534 was increasing in frequency in the overall population. We had hypothesized that within each year, the frequency of Ile1016/Cys1534 would increase faster in the experimental zone receiving insecticide treatment than in the buffer zone even though there was likely to be movement between the two areas. The sample size from the spray zone for the 2013 suppression experiment was relatively small due to the efficacy of the spraying regime, with 252 mosquitoes analyzed from the buffer zone but only 120 analyzed from the spray zone. After spraying, the resistance allele frequency increased in the spray zone but not the buffer zone (Figure 7), rising above the buffer zone by 31% in July (*p* = 0.022) and 64% in August (*p* = 0.0068). In 2014, phenotypic resistance was higher than in 2013. A total of 2178 samples were analyzed for 2014 with a minimum of 52 per group (May, spray zone) and a maximum of 218 per group (October, spray zone) (Figure 7). Prior to the experimental spray event, the allele frequencies in the spray and buffer zones were not significantly different from each other. During the 6‐week intense insecticide selection in the spray zone, the haplotype frequencies of the Ile1016/Cys1534 haplotypes increased in the spray zone relative to the buffer zone by 24% (May, *p* = 0.0011), 19% (July, *p* = 0.0002), and 18% (August, *p* = 0.0091), and finally 11% higher in October (*p* = 0.037) (Figure 7).

## 4 DISCUSSION

We examined the temporal and spatial patterns of *kdr* alleles in *Ae*. *aegypti* from Iquitos, Peru, across an 18‐year period (2000–2017) and identified two episodes of selection for *kdr* haplotypes. We demonstrated that both resistance alleles, Ile1016 and Cys1534, experienced strong selective pressure and that Cys1534 appears to be partially dominant over the wild‐type allele. In addition, we detected significant spatial heterogeneity on the scale of a few city blocks, which supports findings reported elsewhere (Deming et al., 2016; Estep et al., 2018; Grossman et al., 2019). Based on the extensive duration and area of sampling, this is the most thorough study of *kdr* population genetics in *Ae*. *aegypti* to date and it confirms the remarkable capacity for this species to adapt rapidly to insecticidal pressures.

### 4.1 Haplotype dynamics

Three *kdr* alleles coding for Leu410, Ile1016, and Cys1534 that have been shown to contribute to pyrethroid resistance in *Ae*. *aegypti* in Central and South America were detected in Iquitos. Our data support previous conclusions by Saavedra‐Rodriguez et al. (2018) who reported evidence of parallel evolution at loci 410 and 1016. The resistance alleles Leu410 and Ile1016 mostly occurred in the same haplotype and rose simultaneously in frequency in the Iquitos population; therefore, only loci 1016 and 1534 were used for haplotype analyses.

In contrast to the haplotype dynamics observed by Vera‐Maloof et al. (2014) where the allele frequencies of Ile1016 and Cys1534 rose together, in Iquitos, the susceptible wild‐type Val1016/Phe1534 haplotype was initially completely replaced by the Val1016/Cys1534 haplotype. Once that haplotype was at or near fixation, the haplotype carrying two resistance alleles, Ile1016/Cys1534, emerged and quickly rose in frequency, replacing the Val1016/Cys1534 haplotype. This indicates that both resistance haplotypes occurred in Iquitos and that there were likely fitness differences between the two haplotypes.

Temporal results indicate that, prior to the municipal citywide spraying of pyrethroids that commenced in 2002, the majority (>99%) of haplotypes in the population were wild type and <1% of the haplotypes were Val1016/Cys1534 (Figure 3). Individuals carrying the Val1016/Cys1534 haplotype could either represent standing genetic variation in the population due to mutation/selection balance or to historical selection of the population exposed to DDT during the hemisphere‐wide *Ae*. *aegypti* eradication campaign. Although DDT also interacts with the VGSC protein (Brengues et al., 2003; Soderlund & Bloomquist, 1990), it has not been used in Peru since 1994 and may have last been used in Iquitos in the 1970s (A. Morrison, personal communication), which predates the reintroduction of *Ae*. *aegypti* to the country. Thus, it is possible that the source population(s) for the reintroduction of the species already contained *kdr* alleles.

### 4.2 Selection and dominance

Our data provide field‐based evidence that pyrethroid spraying selected for multiple *kdr* haplotypes. This is consistent with previous studies that demonstrated functional pyrethroid resistance caused by *kdr* mutations in the laboratory (Du et al., 2013; Hirata et al., 2014) and other field‐based studies that reported high frequencies of *kdr* mutations in resistant populations (e.g. Brito et al., 2018; Deming et al., 2016; Li et al., 2015; Linss et al., 2014).

Mosquitoes in Iquitos responded quickly to new selection pressure from pyrethroids after 2002 when the Val1016/Cys1534 haplotype rapidly rose to high frequencies. The V1016I substitution was not detected in Iquitos until 2010. The later emergence of the resistance allele at the 1016 locus suggests that this genetic mutation was likely not present in the population prior to 2010. In 2010, Iquitos sprayed α‐cypermethrin for the first time. The specialized chemistry of that particular formulation may have applied a different selection pressure to the population, resulting in an increase in Ile1016/Cys1534 frequency. Alternatively, the mutation may have arrived in Iquitos via immigration from resistant populations, such as neighboring Brazil where the mutation was first detected in 2006 (Linss et al., 2014). While Iquitos is a fairly isolated city with no roads coming into it from other cities, planes and boats arrive daily, which may facilitate mosquito immigration (Guagliardo et al., 2015).

We found evidence of selection pressure in favor of *kdr* mutations. The true selection pressure, however, was likely underestimated because several assumptions of both WFABC and genotype‐frequency models were violated. The selective environment was not constant because the insecticide used, frequency of sprays, and the number of houses sprayed in any given pyrethroid application varied across time and space (Table S1). The model also assumed that the loci are independent, but V1016I and F1534C are tightly linked in the genome. Despite the possible underestimation of selection strength, the estimated mean values indicate strong selection pressure for both resistance alleles.

The Cys1534 resistance allele was estimated to be partially dominant to the Phe1534 susceptible allele by both the WFABC model and our genotype‐frequency model using pMCMC. While some previous investigators found evidence that *kdr* alleles are recessive, they used laboratory, phenotypic exposure assays to determine inheritance (Brito et al., 2018; Saavedra‐Rodriguez et al., 2007). Others suggested an additive effect with a certain combination of *kdr* alleles (Ishak et al., 2015; Plernsub et al., 2016). The WFABC method employed in this paper determined inheritance based on the number of generations that an allele existed at low frequencies and did not include phenotypic data (Foll et al., 2014). Phenotypic resistance can be caused by multiple mechanisms, and while the *kdr* mutations can be useful genetic markers for populations under selective pressure, resistance itself is more complex and involves additional genes and metabolic mechanisms (Smith et al., 2019). In addition, the dominance of an allele can vary depending on environment and genetic background (Bourguet et al., 2000). All of this taken together may explain the estimated differences in dominance between results from this study and those from previous investigations.

The incomplete dominance estimated here by both the WFABC model and our own population genetics model predicted increases in the rate at which the allele frequencies would rise in the population from small frequencies. This is because most resistance alleles were present as heterozygotes, and higher dominance would give greater selective advantage to the heterozygote compared to the homozygous susceptible under spray conditions. Similarly, when the resistance allele was detected at high frequencies, the susceptible allele was present mostly as heterozygotes and quickly fell out of the population (Conner & Hartl, 2004).

While neither the WFABC model nor our own population genetics model accounted for spatial heterogeneity or migration, we know that these phenomena play important roles in *Ae*. *aegypti* population dynamics. Due in part to their limited flight dispersal, this species is thought to have strong spatial structuring. We also know that in the early years of spraying pyrethroids in Iquitos, that the population sizes were significantly reduced (Harrington et al., 2005; Hlaing et al., 2010; Morrison et al., 2008). It is possible that these factors are contributing to the rate of resistance evolution rather than the dominance of the Cys1534 resistance allele. Because the dominance estimate for Ile1016 indicated partial recessivity, future investigations may be able to clarify the roles that spatial heterogeneity, migration, and dominance play in the rate of evolution at these two loci.

### 4.3 Spatial heterogeneity

As has been found in previous studies, we were able to detect heterogeneity in *kdr* frequencies at the citywide scale in Iquitos. This is best illustrated by the haplotype frequency data from 2007 and 2008. There appeared to be a decrease in the frequency of resistance alleles in those years (Figure 3), but this result was likely an artifact of an expanded collection regime and not due to an actual decrease in allele frequency across the city (Figure 4). Mosquitoes were first sampled in the southern part of the city in 2007. At the time, the northern part of the city had a very high frequency of the Val1016/Cys1534 haplotype, but the south still had a large frequency of the wild‐type Val1016/Phe1534 haplotype. Phenotypic susceptibility to at least one pyrethroid in this part of the city during this period was confirmed in 2009 by Lenhart et al. (2020). By 2011, however, this part of the city also had a high frequency of the Val1016/Cys1534 haplotype, indicating that resistance alleles may have been spreading from the port in the north toward the southern and less populated portion of Iquitos. High frequencies of resistance alleles first occurred in the northern part of the city and spread south over a 10‐ to 12‐year period, approximately 120–144 mosquito generations. It is unknown whether these resistance alleles arose independently in Iquitos or were introduced via migration. The Val1016/Cys1534 haplotype already existed within the city prior to widespread pyrethroid selection, and it is possible that the Ile1016/Cys1534 haplotype arose via migration but did not reach a detectable frequency until 2010. Future work should explore the demographic history of mosquitoes carrying these haplotypes.

Another indication that spatial heterogeneity exists in Iquitos is our genotype‐frequency pMCMC results where we observed fewer heterozygotes than predicted by the model between October 2002 and July 2004 (Figure S3). For simplicity, our model assumes a homogeneous, randomly mating population. However, we know that *Ae*. *aegypti* tend not to fly far and migrate on average only 150 m in their lifetime (Harrington et al., 2005). In addition, the mosquitoes included in this analysis were collected over the Northern part of Iquitos, but collections from any given month were typically spatially limited to just a few blocks. Therefore, while the genotype frequencies for this analysis are in Hardy–Weinberg equilibrium for 19 out of 22 months (Table S2), our sampling design was likely to detect existing genetic structure. This is highlighted by the difference between the predicted and observed heterozygote frequencies.

Previous work indicated that the frequency of resistance alleles within an area can be highly heterogeneous; however, prior studies were unable to determine whether the heterogeneity was due to heterogeneity in insecticidal pressures or strong population structure and variation in initial resistance allele frequencies (Deming et al., 2016; Linss et al., 2014). We examined the outcomes from two suppression experiments over 16 and 44 calendar weeks in years 2013 and 2014, respectively. Significant differences were detected between the spray and buffer areas in both years. While initial resistance allele frequencies were higher in 2014 than in 2013, a large divergence in resistance allele frequency was detected following the experimental 6‐week pyrethroid spray treatment, providing further evidence that genetic heterogeneity in *Ae*. *aegypti* can exist at a very fine spatial scale. In the 2013 experiment, trends indicated that resistance allele frequencies increased in the spray zone compared to the buffer zone. The larger confidence intervals were likely due to the small sample size available for analysis during that year. Fewer houses were sampled and the spraying regime was much more effective at reducing population size in 2013, likely due to lower levels of resistance compared to the 2014 sampling period.

## 5 CONCLUSION

This study demonstrated the evolution of two important *kdr* haplotypes in *Ae*. *aegypti* over the course of 18 years in Iquitos, Peru. We document a strong association between citywide pyrethroid spraying and dramatic increases in the frequency of specific *kdr* haplotypes, including two periods when dramatic increases in resistance allele frequency were detected. In our examination of the literature on mosquitoes and other insect pests, we could find no cases where a pest evolved so quickly to so few exposures to low or nonresidual insecticide applications. A combination of high estimated selection coefficients for both *kdr* haplotypes and the partial dominance that the resistance allele, Cys1534, exerted over the susceptible allele when under the selection pressure of pyrethroid insecticides likely contributed to the rapid rate of evolution. Results from spatial suppression experiments showed that *kdr* allele frequencies varied not only at the citywide scale, but also on a fine scale within the city.

Taken together, our results highlight the importance of widespread monitoring of *Ae*. *aegypti* for insecticide resistance from the beginning of first applications. Similarly, it will be especially important to adjust the spatial scale of monitoring efforts to better capture the potential heterogeneity in allele frequencies within an area. The future use of pyrethroids for vector control in Iquitos remains uncertain. Even if the *kdr* resistance haplotype frequencies fall in the absence of pyrethroid selection pressure, resistance is likely to rebound from low frequencies if previously used pyrethroids become used widely once again. Currently, the Ministry of Health is using the organophosphate, malathion, for vector control; however, the results of this study should caution against relying on the use of a single class of insecticides.

In fact, a wide range of traditional and novel techniques are being considered for vector control in the city. Possible strategies include placing more emphasis on larval habit management, using spatial repellents instead of ULV spraying, and targeted residual spraying with novel nonpyrethroids. Genetic‐based control strategies such as Wolbachia for population suppression or replacement and the sterile insect technique are also being considered. It is likely that a combination of methods may need to be utilized to achieve optimal vector control. No matter which solutions that are implemented, resistance management should be a key goal from the beginning.

## CONFLICT OF INTEREST

The authors have no conflicts of interest to declare.

## DISCLAIMER

The views expressed in this manuscript are those of the authors and do not necessarily reflect the official policy or position of the Department of the Navy, Department of Defense, the Centers for Disease Control and Prevention, nor the U.S. Government.

## COPYRIGHT STATEMENT

Some authors of this manuscript are military service members and employees of the U.S. Government. This work was prepared as part of their official duties. Title 17 U.S.C. §105 provides that “Copyright protection under this Title is not available for any work of the United States Government.” Title 17 U.S.C. §101 defines a U.S. Government work as a work prepared by a military service member or employee of the U.S. Government as part of that person's official duties.

## Supporting information

Fig S1

Fig S2

Fig S3

Table S1

Table S2

Appendix S1

## Data Availability

The data that support the findings of this study are openly available in Dryad at https://doi.org/10.5061/dryad.2fqz612pc. The code used for data analyses in this study is available in GitHub at https://github.com/jenbaltzegar/IQTmosq (Baltzegar, 2021).
