# Supplementary figures and images for: Rapid evolution of knockdown resistance haplotypes in response to pyrethroid selection in Aedes aegypti
Source: Evol Appl. 2021 Jul 9;14(8):2098–113. doi: 10.1111/eva.13269 (PMC8372076; doi:10.1111/eva.13269)

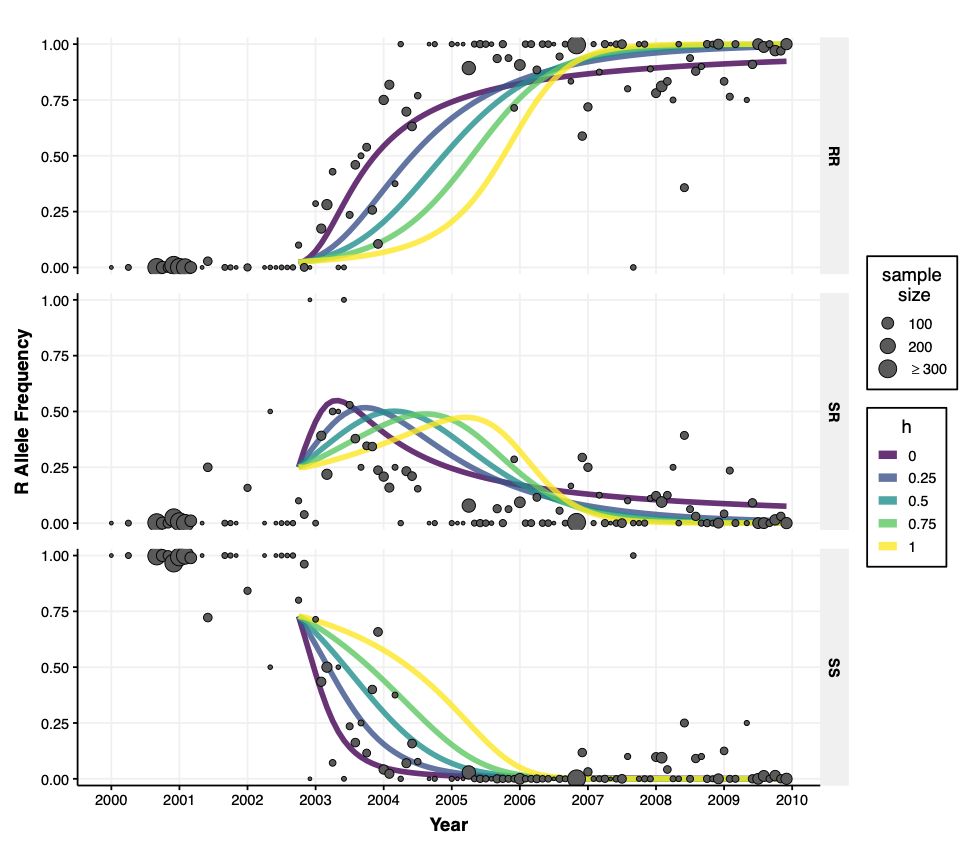

Supplement: Supplementary file 1 — Fig S1 [file EVA-14-2098-s004.png]

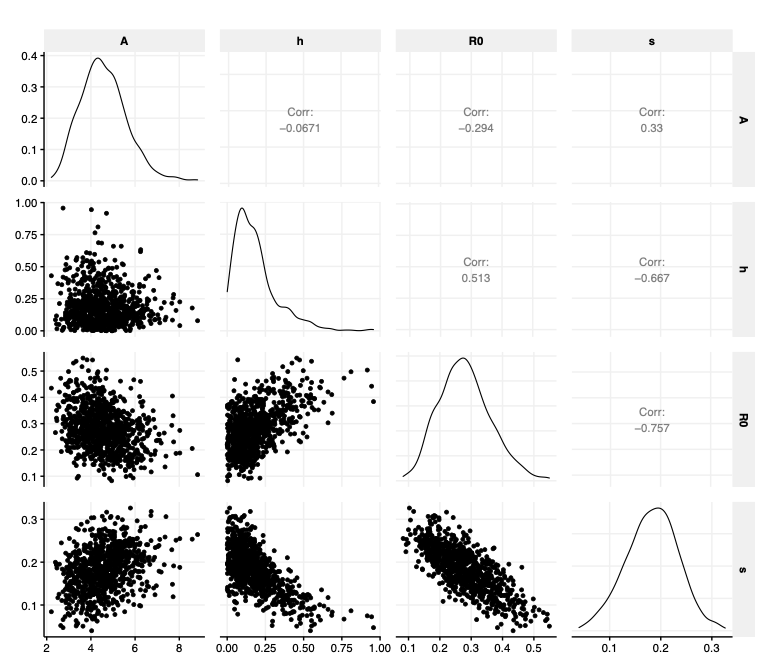

Supplement: Supplementary file 2 — Fig S2 [file EVA-14-2098-s003.png]

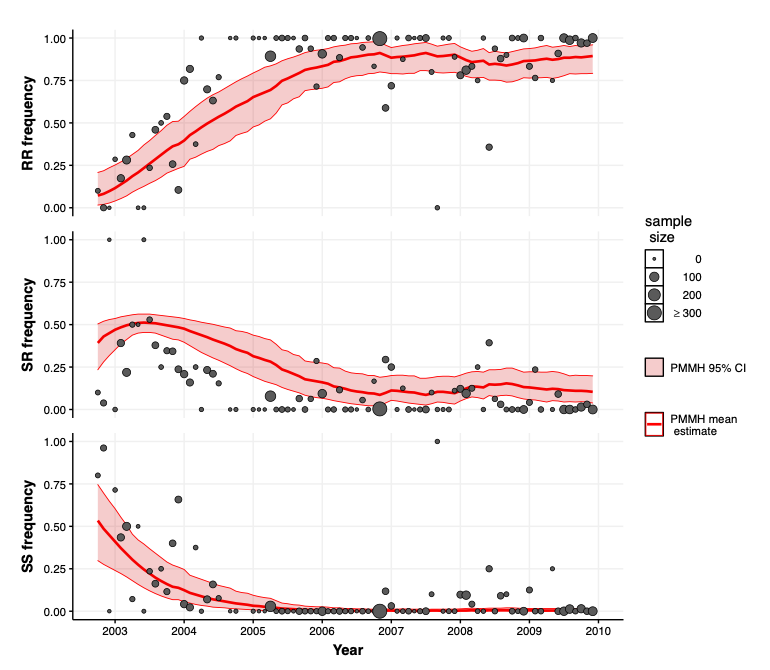

Supplement: Supplementary file 3 — Fig S3 [file EVA-14-2098-s001.png]
