# Supplementary material for: Rapid evolution of knockdown resistance haplotypes in response to pyrethroid selection in Aedes aegypti
Source: Evol Appl. 2021 Jul 9;14(8):2098–113. doi: 10.1111/eva.13269 (PMC8372076; doi:10.1111/eva.13269)

**Supplemental Table 1.** Compiled Ministry of Health insecticide spraying information for Iquitos, Peru from 2000 - 2016. Detailed information about insecticide applications is not available for rows highlighted in grey.


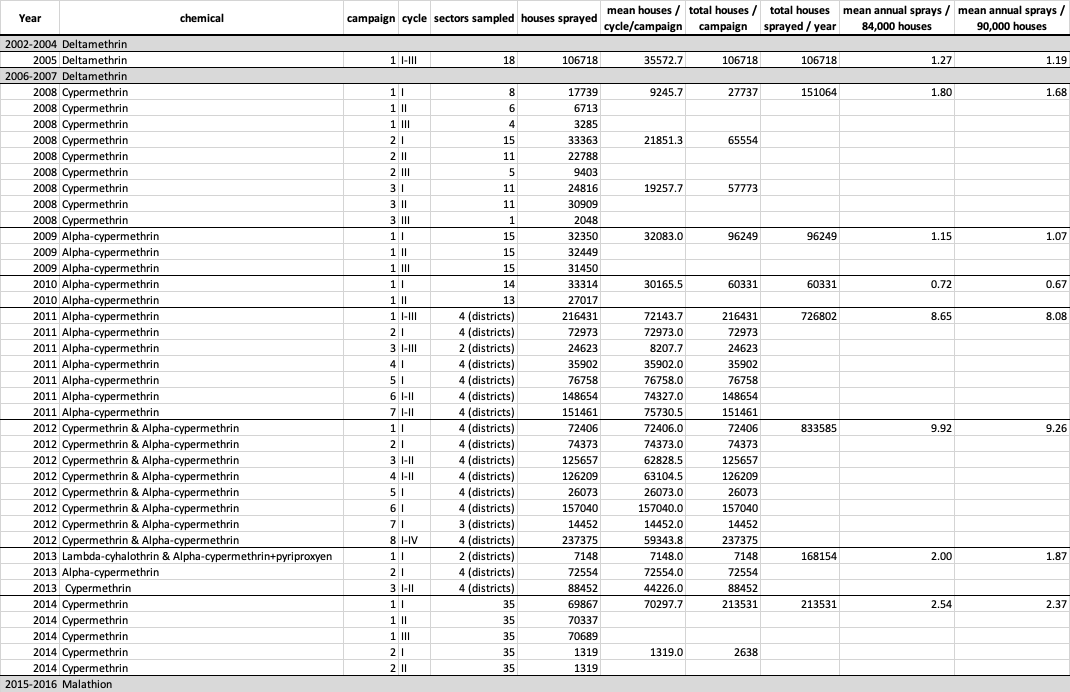

Supplement: Supplementary file 4 — Table S1 [file EVA-14-2098-s006.docx]
