# Supplementary material for: Rapid evolution of knockdown resistance haplotypes in response to pyrethroid selection in Aedes aegypti
Source: Evol Appl. 2021 Jul 9;14(8):2098–113. doi: 10.1111/eva.13269 (PMC8372076; doi:10.1111/eva.13269)
Supplement: Supplementary file 5 — Table S2 [file EVA-14-2098-s002.docx]

**Supplemental Table 2.** Hardy-Weinberg equilibrium (HWE) was calculated at locus 1534 for select months when the resistance allele, Cys1534, was increasing using the ‘genetics’ package in R (v. 3.5.0) (Warnes *et al.,* 2019). The Bonferroni significance level is 2.27 x 10^-3^. Months with significant p-values are marked with an asterisk.

| Date | p-value |
| --- | --- |
| 2002-10 | 1.58E-01 |
| 2002-11 | 1.00E+00 |
| 2002-12 | 1.00E+00 |
| 2003-01 | 6.06E-03* |
| 2003-02 | 3.46E-02 |
| 2003-03 | 4.88E-08* |
| 2003-04 | 1.00E+00 |
| 2003-05 | 1.00E+00 |
| 2003-06 | 4.00E-01 |
| 2003-07 | 1.00E+00 |
| 2003-08 | 1.30E-01 |
| 2003-09 | 1.33E-01 |
| 2003-10 | 1.41E-01 |
| 2003-11 | 8.73E-02 |
| 2003-12 | 7.09E-03* |
| 2004-01 | 7.26E-01 |
| 2004-02 | 6.61E-01 |
| 2004-03 | 1.99E-01 |
| 2004-04 | 3.33E-01 |
| 2004-05 | 3.51E-01 |
| 2004-06 | 2.06E-01 |
| 2004-07 | 2.35E-01 |
