# Supplementary material for: Rapid evolution of knockdown resistance haplotypes in response to pyrethroid selection in Aedes aegypti
Source: Evol Appl. 2021 Jul 9;14(8):2098–113. doi: 10.1111/eva.13269 (PMC8372076; doi:10.1111/eva.13269)
Supplement: Supplementary file 6 — Appendix S1 [file EVA-14-2098-s005.docx]

**Supplemental Materials**

**Supplemental Methods 1.** Detailed description of the genotype-frequency model.

We use a genotype-frequency model to evaluate the spread of the resistance allele at the 1534 locus of the *VGSC*. We assumed the population was well-mixed with random mating and that there were discrete (non-overlapping) generations of mosquitoes, each lasting one month. We also assumed the population was contained, without immigration or emigration of mosquitoes.

We let allele R represent the resistance allele, while allele S represents the wild-type, susceptible allele. The frequencies of each possible genotype were denoted by *X_RR_*, *X_SR_*, and *X_SS_*, with $\vec{X}(t)$ denoting the vector of all three genotype frequencies at generation *t*. For brevity, we omit the vector notation from all vector variables and include a subscript for genotype *i* when referring to a specific element of the vector. The frequency of the R allele in the population is expected to be low until spraying begins because the mutation would likely confer a fitness cost (e.g., proportionately less individuals successfully mate or individuals contribute proportionately fewer offspring) in absence of spraying. For simplicity, we do not consider *de novo* mutations and assume the initial genotype frequencies begin at Hardy-Weinberg equilibrium. Letting *p*(*t*) denote the genotype probabilities of offspring entering generation *t*, we can calculate *p*(*1*) from the starting allele frequency of the resistance allele, *R_0_*:

$$p_{RR}(1)={R_{0}}^{2},p_{SR}(1)=2R_{0}\left( 1-R_{0} \right), \mathrm{and} p_{SS}(1)=\left( 1-R_{0} \right)^{2}.$$

While there is spraying, we assume mosquitoes with an S allele suffer a loss in fitness. Assuming RR is the favored genotype while under selection, SS and SR individuals have a proportionally smaller contribution to the mating pool due to death or fewer offspring. The fitness cost to SS individuals (percent decrease of SS relative to RR) is *s*, and the fitness cost to SR individuals is *h*$\cdot$*s*, where *h* is the dominance of the cost to the S allele.

Using the current frequencies, *X*(*t*), the genotype probabilities of the next generation, *p*(*t*+1), can be calculated based on the probabilities of each of the six possible matings of genotypes. The assumption of a well-mixed and randomly mating population allows the probability of a given mating to be calculated as the product of the pair of genotype frequencies. Each pairing of different genotypes is multiplied by a factor of two to account for the two possible couplings of males and females. The offspring probabilities from each mating are calculated assuming Mendelian inheritance. Fitness costs, as described above, result in a relative reduction in the SR and SS frequencies. The resulting set of difference equations to calculate *p*(*t*+1) from *X*(*t*) (omitting dependence on time for brevity) is:

$$p_{RR}=\left( {X_{RR}}^{2}+{{0.25X}_{SR}}^{2}+X_{RR}X_{SR} \right)\frac{1}{\bar{w}}$$

$$p_{SR}=\left( 1-hs \right)\left( {2X}_{RR}X_{SS}+X_{RR}X_{SR}+X_{SS}X_{SR}+{{0.5X}_{SR}}^{2} \right)\frac{1}{\bar{w}}$$

$$p_{SS}=\left( 1-s \right)\left( {X_{SS}}^{2}+{{0.25X}_{SR}}^{2}+X_{SS}X_{SR} \right)\frac{1}{\bar{w}},$$

where the mean fitness, $\bar{w}$, is the sum of the un-normalized frequencies and normalizes so the next generation frequencies sum to 1.

Temporarily ignoring the effects of genetic drift, we can assume the next generation frequencies *X*(*t*+1) are equal to the expected probabilities *p*(*t*+1)*.* By also assuming that the samples are independent and taken at random spatially, maximum likelihood estimation can be used. The data vector from each generation t, $Y(t)$, consists of counts for each genotype, and we denote the sample size, which varies by generation, by $N_{s}(t)$. By temporarily assuming that samples of individual mosquitoes in a given generation are independent and identically distributed, the sampling distribution of $Y(t)$ can be approximated by a multinomial distribution with probabilities $X(t)$ as predicted from simulating the full time series with parameters $\theta=(s,h,R_{0})$. The likelihood, L, and log-likelihood, $l$, of the data at time t are given by:

$$L\left( Y\left( t \right)|\theta\right)=\frac{N_{s}\left( t \right)!}{Y_{RR}\left( t \right)!Y_{SR}\left( t \right)!Y_{SS}\left( t \right)!}{X_{RR}\left( t \right)}^{Y_{RR}\left( t \right)}{X_{SR}\left( t \right)}^{Y_{SR}\left( t \right)}{X_{SS}\left( t \right)}^{Y_{SS}\left( t \right)}$$

$$l\left( Y\left( t \right)|\theta\right)=c_{1}+Y_{RR}\left( t \right)\log\left( X_{RR}\left( t \right) \right)+Y_{SR}\left( t \right)\log\left( X_{SR}\left( t \right) \right)+Y_{SS}\left( t \right)\log\left( X_{SS}\left( t \right) \right) .$$

Here, *c_1_* involves terms that do not have a dependence on *X*. Parameter estimates then maximize the log-likelihood of all samples over time for all *T* generations based on the assumption that samples in each generation are independent:

$$l\left( Y|\theta\right)=c_{2}+\sum_{t=1}^{T} \sum_{i=1}^{3} Y_{i}\left( t \right)\log\left( X_{i}\left( t \right) \right)\left( t \right)$$

Here, *c_2_* involves terms that do not have a dependence on *X.*

We incorporated stochasticity from genetic drift by assuming a mosquito count equal to the effective population size, *N_e_*, in each generation, assumed to be 500 as in the main text. We let the genotype counts in the next generation, *C*(*t+*1), be distributed multinomially based on the genotype probabilities *p*(*t*). Then we have:

$$C\left( t+1 \right)\sim Multinomial(n=N_{e},prob=p(t))$$

$$X\left( t+1 \right)= \frac{C(t+1)}{N_{e}}.$$

We can additionally loosen our assumption that samples in a given generation are independent and identically distributed. We keep our assumption that the sampling distribution at generation *t* is independent of sampling in previous generations, meaning there is only dependence on the frequencies in that generation, *X*(*t*). We also assume that while sampling removes mosquitoes from the population, the resulting effect on the population genetics is small enough to ignore. To account for the possibility of multiple mosquitoes being sampled from the same house, and heterogeneity in genotype frequencies we employ an overdispersed sampling distribution with added variance compared to a multinomial distribution. Specifically, we employ the Dirichlet-multinomial distribution parameterized by *AX(t)*, where *A* is an overdispersion parameter, which gives the following probability density of *Y* :

$$f\left( Y=y | X,A \right)=\frac{N!\Gamma(A)}{\Gamma(N_{s}+A)}\prod_{i=1}^{3} \frac{\Gamma(y_{i}+AX_{i})}{y_{i}!\Gamma(AX_{i})},$$

where $\Gamma(\cdot)$ is the gamma function. Smaller values of *A* result in higher sample variance, essentially accounting for the possibility of drawing a large fraction of the total samples from few individual houses, which may have mosquito genotype frequencies much different from that of the entire city. To allow for calculation of likelihoods when $X_{i}(t)=0$ but $Y_{i}(t)>0$, in practice we parameterize the distribution with *AX* + 0.0001. This has a minimal effect on the properties of the distribution but accounts for the possibility of additional sampling error, e.g., if a sample was mislabeled.

The full population genetics model and sampling distribution form a Hidden Markov Model (HMM). We conduct Bayesian inference on the HMM using particle Markov chain Monte Carlo (pMCMC). We implement pMCMC with a multivariate normal proposal distribution and adaptive Metropolis-Hastings acceptance in R (R Core Team 2019) using the package nimble (de Valpine *et al.,* 2017). We used 1000 particles for the particle filter. For parameters in the range [0,1] (*s*, *h*, and *R_0_*), we use uninformative priors of Beta(1,1). For *A*, we use the uninformative prior $A\sim Gamma(0.01,0.01).$ To improve the time to convergence, we initialize the parameters using their maximum likelihood estimates. The output of pMCMC is the joint posterior distribution of the parameters along with samples of full time series of genotype frequencies, which we used to construct 95% credible intervals of genotype frequencies.
